# Research on atmospheric temperature change and its adaptive strategies in winter wheat and summer maize dual-cropping region of North China Plain during 1961–2021—A case study of Jiaozuo City in Henan Province

**DOI:** 10.1371/journal.pone.0325781

**Published:** 2025-06-27

**Authors:** Chengyuan Hao, Sunan He

**Affiliations:** 1 School of Surveying and Land Information Engineering, Henan Polytechnic University, Jiaozuo, Henan, China; 2 School of Resources and Environment, Henan Polytechnic University, Jiaozuo, Henan, China; The Chinese University of Hong Kong, HONG KONG

## Abstract

Global warming has caused the frequency and intensity of extreme weather events in agriculture. Therefore, research on temperature changes in major grain-producing areas is essential for formulating adaptation strategy. This article takes Jiaozuo City, Henan Province, China as the research area. Based on daily maximum, minimum and mean temperatures from seven meteorological stations for the period 1961**–**2021, the comprehensive indications of temperature changes were analyzed using the linear tendency estimation, Mann-Kendall test, and levels fluctuation. Fourteen climate indices in four classes, including the mean temperature index, absolute extreme temperature index, relative extreme temperature index, and disaster temperature index, were applied. The results indicated four aspects. Firstly, the average regional warming speed was 0.259°C/10a**—**slightly lower than the national average, and the years of the highest and lowest values were 2021 and 1984, respectively. Secondly, the daily minimum and maximum temperatures increased significantly, which were 0.395°C/10a and 0.200°C/10a respectively**—**less than the national mean. The relevant low temperature index showed proper decreasing trend while the diurnal range of annual extreme temperature showed fluctuating**—**decreasing first and then increasing. Thirdly, the relevant e high temperature indices of plain urban area were larger while the relevant low temperature indices of mountain hilly area were smaller. The relative high temperature indices showed an increasing trend while the relevant low temperature indices tended to decrease. Fourthly, high temperature disasters generally declined before the 1980s and increased thereafter; low temperature disasters showed a decreasing trend overall. This study suggests that in the two-cropping regions of winter wheat and summer maize, attention should be paid to the increasing trend of high-temperature days. Additionally, heat-tolerant varieties should be cultivated to expand the planting area of maize for adapting to increasing drought disasters induced by high temperature, as well as to establish an agricultural disaster-risk mechanism for addressing high temperature meteorological disasters.

## 1 Introduction

The global risks report 2022 released by the World Economic Forum (2022) indicated that climate change and extreme weather are the paramount current global risks, and multiple climatological events have evidenced climate change—mainly characterized by rising temperatures [1,2]. Reports 3^rd^ to 6^th^ of the Intergovernmental Panel on Climate Change (IPCC for short) have all indicated progressively stronger warming trends [3,4]. In other words, each assessment shows warmer temperatures than recorded the previous year, indicating an increasingly global warming trend in recent years [5]. However, temperature increase is not uniform. A basic consensus has been reached that this regional heterogeneity warming is not only reflected in the increase of the daily mean temperature, but also in the increase of the relative temperature index represented by the numbers of summer days and hot nights [6]. Specifically, disaster temperature events are becoming increasingly frequent and concentrated [7,8], especially in the winter wheat and summer maize dual-cropping region of China [9,10]. This region is characterized by high cropping intensity, water-sensitive growth periods, and frequent exposure to climatic extremes. It is especially vulnerable to high-temperature events due to the overlap between key crop developmental stages (e.g., wheat grain filling and maize seedling stages) and the occurrence of extreme heat in late spring and summer. Prolonged high temperatures during these periods can severely impact crop yields, disrupt phenological development, and increase the risk of drought stress. Therefore, ascertaining temperature trends and extreme events in this region is critical for ensuring food security and developing targeted adaptation strategies.

Currently, research on regional response to global warming is at the forefront of integrated physical geography [11,12]. Extreme temperature events adversely impact food production, human settlements, and human health [13–15]. Therefore, numerous studies—domestic as well as foreign—have explored this issue from multiple research perspectives and expression modes. The variation trend and distribution pattern of temperature—especially, extreme temperature—are impacted by natural conditions and human activities, and therefore, differ across regions. However, China has a vast territory with immense regional differences in both natural and cultural conditions—for example, there are sparsely populated regions as well as densely populated ones, such as the North China Plain [16,17]. Particularly, research on long-term temperature change in the North China Plain has been mostly conducted on a provincial scale—in the Hebei, Shandong, Henan, Jiangsu, and Anhui Provinces. Generally, cogent city-scale research has been rather rare while the spatial distribution of meteorological stations used in provincial scale research has been mostly scattered [18,19]. For example, Yin et al. [20] had selected the daily temperature data of 13 meteorological stations from 1951 to 2013 to study the trend and probability characteristics of extreme temperature in 1.07 × 10^5^ km^2^ of the Jiangsu Province, including minimum and maximum temperatures. Hao et al. [21] utilized the climate changing tendency rate to express the temporal and spatial variation characteristics of both extreme minimum and maximum temperatures in 1.89 × 10^5^ km^2^ of the Hebei Province, but no mean temperature, based on the daily extreme data of 50 meteorological stations from 1961 to 2008. Detailed research exists in this area. For instance, Yu et al. [22] calculated six relative temperature indices based on the daily temperature data of 14 meteorological stations from 1971 to 2013, and conducted the characteristics analysis of the spatio-temporal variation on extreme temperature in 1.56 × 10^5^ km^2^ of the Shandong Province. Liu et al. [23] comprehensively applied a variety of mathematical techniques such as the climate tendency rate, sliding T-test, and cross wavelet to explore the characteristics of temperature change under multiple time-scales in 1.40 × 10^5^ km^2^ of the Anhui Province, based on the climate data of 17 meteorological stations from 1960 to 2015. More notably, for 1.67 × 10^5^ km^2^ of the Henan Province, Wang [24] applied the linear tendency estimation and M-K test to investigate the spatio-temporal variation of extreme temperature based on the daily temperature data of 32 meteorological stations for the period 1961–2011. Zhao et al. [25] analyzed the changing process of the cold and warm temperature indices based on the basic data of 17 national basic/benchmark meteorological stations. Nevertheless, research has typically focused on either a limited number of extreme temperature indices or emphasized general climatic trends; a comprehensive analysis of multiple types of temperature indices using an integrated framework remains scarce. Moreover, few studies have explicitly linked temperature dynamics to agricultural relevance, particularly in terms of identifying adaptive strategies at the local level to cope with climate-induced risks.

To address these gaps, this study focuses on Jiaozuo City in the Henan Province, a representative zone of the North China Plain and one of China’s key grain-producing regiones. Unlike previous provincial-scale analyses, this study utilizes daily temperature data from seven densely distributed meteorological stations spanning the period 1961–2021 to investigate the long-term spatiotemporal patterns of temperature change at the city scale. Fourteen temperature indices across four categories—mean, extreme, relative, and disaster-related—are analyzed based on linear trend estimation and anomaly analysis. Moreover, this study not only identifies regional temperature change patterns but also explicitly links them to agricultural impacts, proposing targeted adaptive strategies for crop production under climate warming. The present research not only addresses the knowledge gap in the analysis of temperature trends at the urban scale in the Huang-Huai-Hai Plain, but also applies multiple indicators to conduct a more comprehensive and integrated evaluation of temperature changes. By proposing agricultural adaptation measures with regional characteristics, the practical relevance of climate research has been enhanced. This academic endeavor enhances the practical relevance of climate research by proposing region-specific agricultural adaptation measures. The research results can not only provide data support for the rational utilization of climate resource to vigorously develop sustainable agriculture, but also offer theoretical evidence for local governments to make decisions regarding agricultural disaster reduction and relief [26].

## 2 Regional natural profile and research data

### 2.1 Regional natural profile

**Fig 1:**
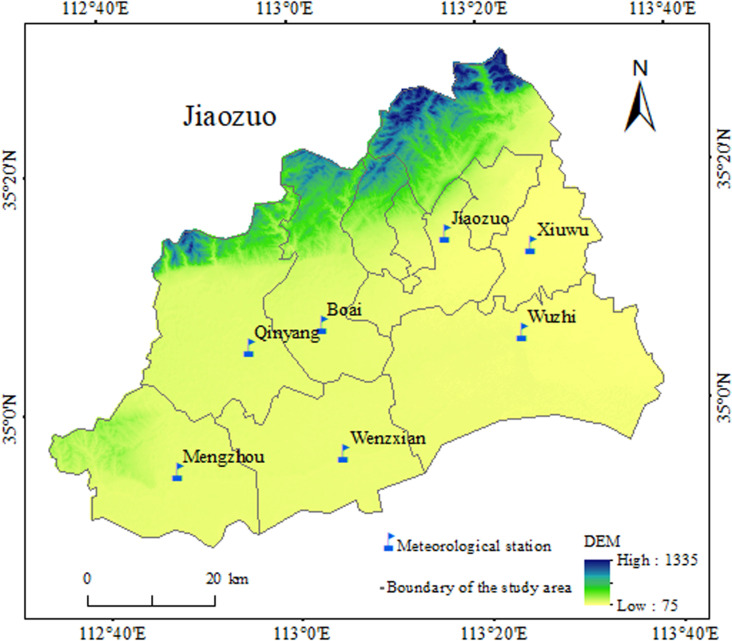
Study area location and meteorological stations distribution.

Jiaozuo City is located in the north of the Henan Province, between the Taihangshan Mountains in the north and the Yellow River in the south, with the longitude and latitude range of 112°35′31″E to 113°38′35″E and 34°49′03″N to 35°29′45″N, respectively, and total area of 4071 km^2^ (Fig 1). In terms of regional topography or geomorphology, the northern part of the research region is mountainous, with the highest elevation of 1308 m, accounting for 25.17% of the total area. The southern part comprises alluvial plain, with the lowest elevation of 90 m, accounting for 74.83%. In terms of regional climate, Jiaozuo City belongs to the warm temperate continental semi-humid monsoon climate type. It has distinct four seasons: spring is warm and rainy, summer is hot and rainy, autumn is cool and dry, and winter is cold with a little snow. The average annual precipitation is 586.7 mm, and the summer rainfall from June to August accounts for approximately 60% of the total annual rainfall. The annual daily temperature is 14.839 °C while the annual accumulated temperature of ≥10 °C is 4874.8 °C. In terms of regional vegetation and soil, zonal vegetation type is warm temperate deciduous broad-leaved forest while zonal soil is tidal soil and brown soil. Tidal soil is mainly distributed in the southern alluvial plain or valley zone with an altitude of less than 1000 m, while brown soil is primarily distributed in the northern mountainous region and piedmont inclined plain.

### 2.2 Data sources

Jiaozuo City consists of seven administrative units. Therefore, the research region includes seven meteorological stations: the Qinyang, Boai, Shanyang, Xiuwu, Wuzhi, Mengzhou, and Wenxian Stations (hereinafter referred to as Qinyang, Boai, Shanyang, Xiuwu, Wuzhi, Mengzhou, and Wenxian). The daily temperature data consist of three types: the daily mean temperature, daily minimum temperature, and the daily maximum temperature, all obtained from the National Meteorological Information Center of the China Meteorological Administration. The time period is 61 years, from January 1, 1961 to December 31, 2021. It is noteworthy that in late July in 2021, a once-in-a-century severe flood occurred in the research region. Based on preliminary statistical analysis, precipitation and temperature during 2021 and multi-year for the period 1961–2021, and the basic data of the seven stations are presented in Table 1.

**Table 1:** Basic information of related meteorological stations.

Name	Longitude/°C	Latitude/°C	Elevation/m	The year 1961–2021				The year 2021		
				Average annual precipation/mm	Average daily temperature/°C	Extreme high temperature(year)/°C	Extreme low temperature(year)/°C	Annual precipation/mm	Above the average/%	Daily temperature/°C
**Qinyang**	112.92	35.09	120.4	549.5	14.94	43.4(2009)	−17.6(1971)	1236.8	125.07	16.09
**Boai**	113.05	35.12	130.3	574.0	14.79	42.5(2011)	−17.9(1969)	1204.3	109.81	16.70
**Shanyang**	113.27	35.24	113.2	549.2	15.59	43.5(2009)	−17.8(1990)	1407.4	156.26	17.37
**Xiuwu**	113.42	35.22	86.3	560.4	14.61	43.5(1964)	−19.9(1971)	1335.9	138.38	15.69
**Wuzhi**	113.40	35.10	96.1	575.1	14.70	43.6(1966)	−22.4(1990)	1157.2	101.22	16.32
**Mengzhou**	112.79	34.92	117.6	586.9	14.56	42.9(2014)	−17.9(1969)	1290.5	119.88	15.77
**Wenxian**	113.08	34.94	108.5	552.4	14.69	43.2(1966)	−17.0(1969)	1255.1	127.21	16.33

### 2.3 Data expression

Based on Climate Variability and Predictability—one of the six research projects in the World Climate Research Program, and authoritative like the World Meteorological Organization and IPCC—domestic and foreign researchers have variously indicated the temperature changing situation [27–29]. This study, mainly referring to You et al. [30], selected 14 temperature indices in four categories to characterize the comprehensive indicators of temperature change in Jiaozuo City, after conducting a preliminary study of the daily mean, daily minimum, and daily maximum temperature trends of seven stations over 61 years. Particularly, both extreme low temperature ≤ −10 °C and extreme high temperature ≥40 °C are not conducive to agriculture in northern China, including the research region [31]. The specific meanings of temperature indices mentioned above are presented in Table 2.

**Table 2:** Connotations of different temperature indices and their specific meaning.

Sorts of index	Indaicator name	For short	Definitions	Unit
**Mean Temperature Index**	Mean Temperature Mean	TMm	Average value of daily mean temperature	°C
	Maximum Temperature Mean	TMx	Maximum value of daily mean temperature	°C
	Minimum Temperature Mean	TMn	Minimum value of daily mean temperature	°C
**Extreme Temperature Index**	Mean Temperature Maximum	TXm	Average value of daily maximum temperature	°C
	Maximum Temperature Maximum	TXx	Maximum value of daily maximum temperature	°C
	Mean Temperature Minimum	TNm	Average value of daily minimum temperature	°C
	Minimum Temperature Minimum	TNn	Minimum value of daily minimum temperature	°C
	Diurnal Temperature Range	DTR	Mean difference between TX and TN	°C
**Relative Temperature Index**	Summer Days	SU25	Annual count when daily maximum>25 °C	days
	Ice Days	ID0	Annual count when daily maximum<0 °C	days
	Tropical Nights	TR20	Annual count daily minimum>20 °C	days
	Frost Days	FD0	Annual count when daily minimum<0 °C	days
**Disaster Temperature Index**	High Temperature Disaster	HTD40	Annual count when daily maximum≥40 °C	days
	Low Temperature Disaster	LTD-10	Annual count when daily minimum ≤ −10 °C	days

After rigorous quality control and homogenization correction of daily temperature data, this research obtained 14 indices in the four categories mentioned above. When analyzing the time series variation trend of the seven stations in Jiaozuo City, the linear trend method is applied to fit the variation trend of each index, and the Mann-Kendall non-parameter test is conducted to assess the significance of the variation trend, including the temporal-spatial features and the changing or variation conditions of the four indices mentioned above. The data in this article are averaged over all stations, unless specified otherwise, and the study period is January 1, 1961–December 31, 2021, if no specific time period is restricted.

## 3 Results and analysis

### 3.1 Temperature changes based on the mean temperature index

#### 3.1.1 Temporal-spatial features.

**Fig 2:**
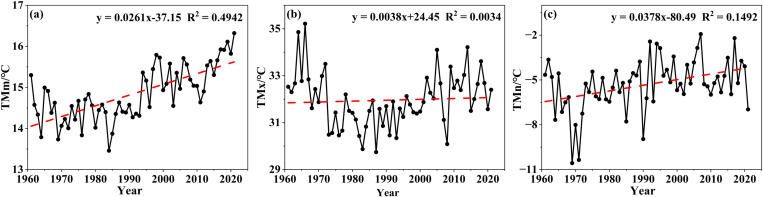
Variation curve of the mean temperature index.

The average values of TMm, TMx, and TMn were 14.84 °C, 31.96 °C, and −5.32 °C, respectively. Correspondingly, TMm showed the most obvious increasing trend with an overall growth rate of 0.259 °C/10a while both TMx and TMn had strong volatility and poor trend (Fig 2). In terms of TMm between the seven stations, the annual average of Shanyang was the highest, reaching 15.59 °C, while that of Mengzhou was the lowest—merely 14.56 °C; there was a difference of more than 1 °C between them. For almost all stations, with the exception of Shanyang, the year of the lowest value was 1984. The maximum TMm of all stations was in 2021, which was from 7.72% to 12.94%—higher than the average annual temperature (Table 1); that is, the annual regional average temperature in 2021 was 10% higher than the average annual temperature. The annual precipitation of all stations in 2021 ranged from 101.22% to 156.26% higher than the average annual precipitation—that is, the regional average precipitation in 2021 was 2.25 times the regional annual precipitation. Briefly put, the year 2021 was not only a once-in-a-century flood year in this research region, but also the year of the highest average temperature during the period studied.

#### 3.1.2 Changing or variation conditions.

**Fig 3:**
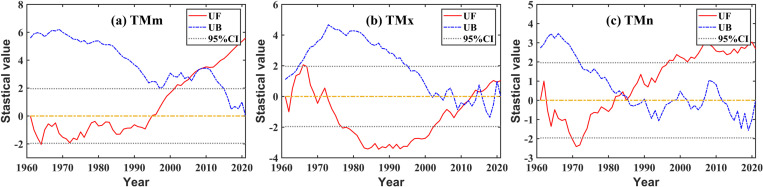
Mann-Kendall test for the mean temperature index.

**Fig 4:**
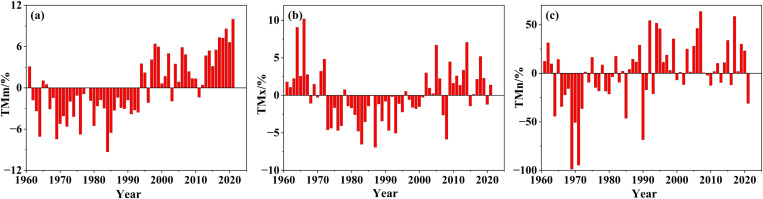
Fluctuation trend of the mean temperature index.

Combined with Fig 3, there are mainly two points of analysis for the fluctuated conditions of the mean temperature index. First, being bounded by 1994, the TMm value of the front part was basically higher while that of the rear part was lower. So, the multi-year trend of TMm was the most regular, with a fluctuation state of first decreasing and then increasing. At the 10-year scale, the average temperature of each decade was 14.47 °C, 14.36 °C, 14.31 °C, 14.98 °C, 15.21 °C, and 15.54 °C, respectively, with a significant increasing trend in the last 40 years, that is, since the 1980s (Fig 4a). Second, divided into three periods based on 1973 and 2002, the TMx values of the two periods both before 1973 and after 2002 were basically higher, while that in the intermediate period was basically lower (Fig 4b); TMx was the most volatile. Although the index of TMn was not as significant as that of TMm, it also indicated a certain increasing trend (Fig 4c); no obvious mutations occurred in TMm, TMx, and TMn.

### 3.2 Temperature changes based on the extreme temperature index

#### 3.2.1 Temporal-spatial features.

**Fig 5:**
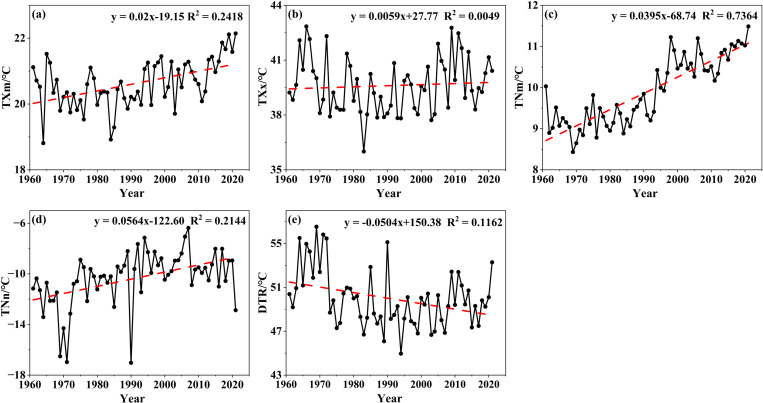
Variation curve of the extreme temperature index.

In terms of multi-year daily maximum temperature, the average of TXm was 20.62 °C with an increasing rate of 0.200 °C/10a (Fig 5a), while the average of TXx was 39.61 °C and the variation tendency was not distinct (Fig 5b). In terms of multi-year daily minimum temperature, the average of TNm was 9.90 °C with an increasing rate of 0.395 °C/10a (Fig 5c), while there was an increasing tendency of TNn but poor trend, which failed to pass the significance test, and the average of TNns was −10.36 °C (Fig 5d). Regarding DTR, there was only a decreasing tendency, but it did not pass the significance test, either (Fig 5e). However, it was noteworthy that the average of 61 years had reached 49.97 °C, and the extreme in 1969 was 56.53 °C. At Mengzhou in 1969, the annual minimum and maximum temperatures were −17.9 °C and 41.1 °C, respectively, with the DTR of 59 °C. The highest DTR of 60.4 °C was in Wuzhi in 1990, with the minimum temperature of −22.4 °C and maximum temperature of 38.0 °C, respectively.

#### 3.2.2 Changing or variation conditions.

**Fig 6:**
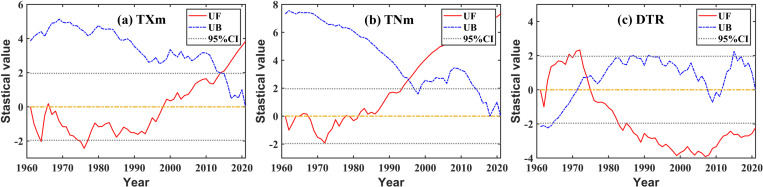
Mann-Kendall test for the extreme temperature index.

**Fig 7:**
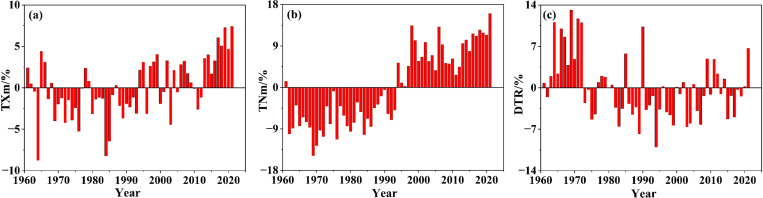
Fluctuation trend of the extreme temperature index.

Regarding the changing condition of TXm for many years, combined with Fig 6, we identified that 1994 was the line of demarcation, roughly: lower before, and then higher (Fig 7a), and there are no obvious mutations. The annual average daily maximum temperatures from 1960s to 2010s were 40.38 °C, 39.41 °C, 38.44 °C, 39.07 °C, 40.02 °C, and 40.23 °C respectively, which decreased first and then increased. Moreover, the fluctuation state of TNm was roughly close to the changing process of TXm mentioned above (Fig 7b), and there are no obvious mutations. The annual average daily minimum temperatures from the 1960s to the 2010s were −12.34 °C, −11.30 °C, −10.77 °C, −9.09 °C, −8.96 °C, and −9.47 °C, respectively, with a generally warming trend, but with a slight fluctuation in the 2000s. Meanwhile, an obvious mutation occurred in 1972; we identified that the growth rate was slower and the fluctuation was smaller than the previous year’s, and later on, the growth rate accelerated and the fluctuation increased. From the 1960s to the 2010s, the DTRs were 52.72 °C, 50.71 °C, 49.22 °C, 48.16 °C, 48.98 °C, and 49.70 °C, respectively, with the fluctuation first decreasing and then increasing (Fig 7c).

In conclusion, regarding the changing process of TXm, TNn, and DTR, there exists a certain law of time sequence regularity. According to that law, the fluctuation state of TXm and DTR first decreased and then increased, while TNm showed an obvious increase. However, neither TXx nor TNn showed a temporal trend.

### 3.3 Temperature changes based on the relative temperature index

#### 3.3.1 Temporal-spatial features.

**Fig 8:**
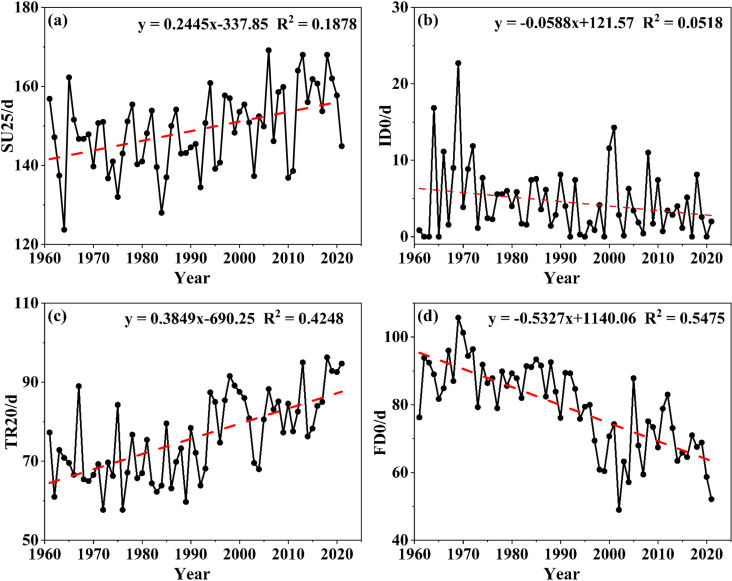
Variation curve of the relative temperature index.

On the one hand, the regional differentiations of both SU25 and ID0 are relatively minor, based on the daily maximum temperatures. First, for SU25, the multi-year average of the whole research region was 148.91d, with 169.14d being the highest value in 2006, and 123.7d being the lowest value in 1964; so, the difference was merely 20.38% (Fig 8a). In terms of the extreme values comparison among the seven stations, the highest SU25 was 174d at Shanyang in 2006, and the lowest value being 120d at Boai in 1964, with a difference of merely 45.00%. In terms of the multi-year mean values comparison among the seven stations, 152.66d at Shanyang was the largest while 146.38d at Boai was the smallest, and the difference between them was merely 4.29%. As depicted in Fig 8b, for ID0, the mean value of seven stations was 4.56d, and the largest value was 22.71d in 1969—especially, the values of both Qinyang and Boai had reached 24d. The lowest value was 0, and there were eight years: 1962, 1963, 1965, 1992, 1995, 1999, 2017, and 2020. The regional comparison of the multi-year mean value among all the stations showed that 4.77d at Wenxian was the largest and the 4.29d at Wuzhi was the smallest; their difference was merely 11.20%.

On the other hand, the regional differentiations of both TR20 and FD0 were relatively large, based on the daily minimum temperatures. First, the multi-year mean of TR20 was 76.05d, with 96.29d in 2018 being the highest, and 57.71d in 1976 being the lowest; so, the difference was 66.85% (Fig 8c). Among the seven stations, the highest was 121d at Shanyang in 2020, and the lowest was 47d at Mengzhou in 1989, with a difference of 157.00%. The regional comparison of multi-year mean value showed that 88.46d at Shanyang was the largest while 70.66d at Mengzhou was the smallest, and their difference was 25.19%. Second, the mean FD0 of all seven stations was 79.39d, with the largest of 105.71d in 1969 and the smallest of 49.00d in 2002; their difference was 116.00% (Fig 8d). In terms of the FD0 extreme values comparison among the seven stations, the highest was 111d at both Qinyang and Boai in 1969 while the lowest was 32d at Shanyang in 2021, with a difference of 247.00%. In terms of the FD0 multi-year mean values comparison, 85.10d at Xiuwu was the largest while 66.89d at Shanyang was the smallest, with a difference of 27.22%.

More importantly, as the largest population agglomeration region in the research region correspondingly, the Shanyang administrative region had the highest SU25 and TR20 values, regardless of the multi-annual mean and annual statistical extreme values. Additionally, the FD0 and ID0 of both Boai County and Qinyang City were the highest, which could be representatives of the northern mountainous region as they were ranked the first and the second, respectively, in altitude. All in all, there were two main conclusions about the relative temperature indices based on the above analysis. First, the regional differentiation of both SU25 and ID0 was smaller while that of both TR20 and FD0 was larger. Second, the relative indices of high temperature in plain urban area were increasing while those of low temperature in mountainous hilly area were decreasing.

#### 3.3.2 Changing or variation conditions.

**Fig 9:**
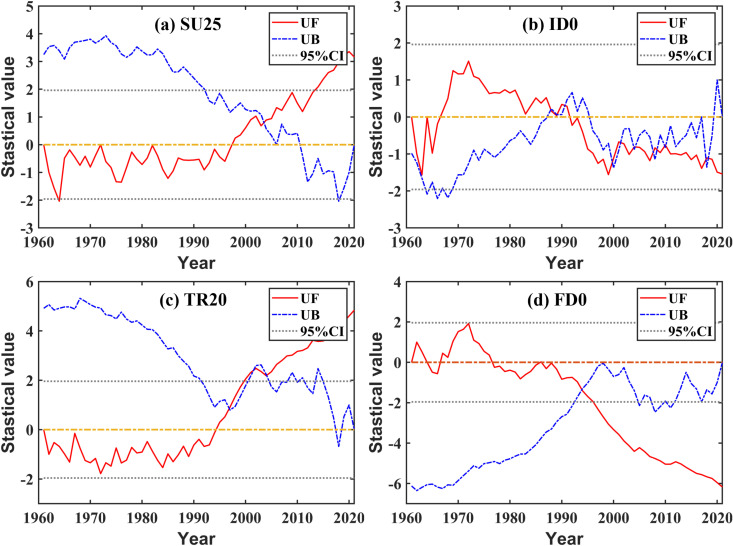
Mann-Kendall test for the relative temperature index.

**Fig 10:**
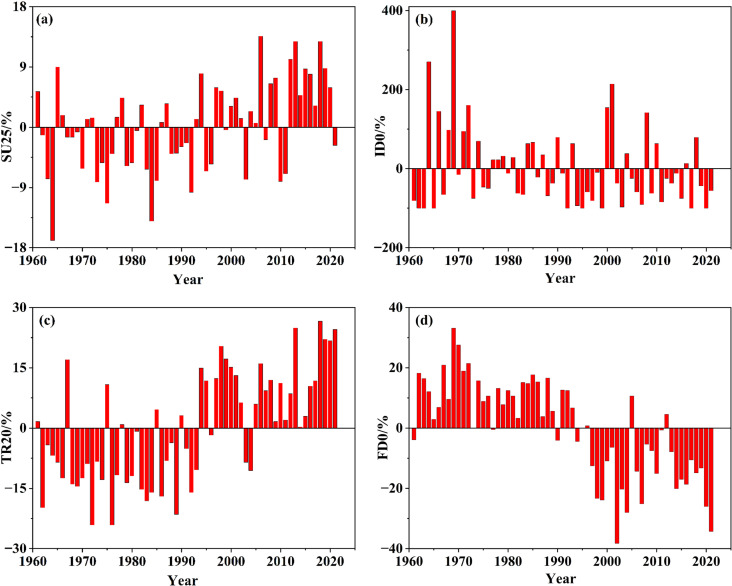
Processes and fluctuating ranges of the relative temperature index.

Combined with Fig 9, irrespective of SU25 and ID0 based on the daily maximum temperature, or TR20 and FD0 based on the daily minimum temperature, the fluctuation trend states were similar, and the fluctuation pattern during the time period before and after 1994 were relatively distinct (Fig 10). However, there were two noteworthy points regarding the changing process or trend characteristics. First, the fluctuation patterns of both SU25 and TR20 indicating high temperature were extremely similar. The values before 1994 were mostly lower while the values after 1994 were mostly higher, showing an increasing trend (Fig 10a and 10c). There is a definite increase of TR20 by degrees, and the average values of intergeneration during the studied period were 70.41d/a, 68.16d/a, 69.00d/a, 80.05d/a, 80.34d/a, 86.04d/a, especially during the last 40a since the 1980s. Second, the fluctuation state of both ID0 and FD0 indicating low temperature was basically the same, and the variation tendency was declining (Fig 10b and 10d). The fluctuation regularity of FD0 was more significant, and the average values of intergeneration in the studied period were 70.41d/a, 68.16d/a, 69.00d/a, 80.05d/a, 80.34d/a, and 86.04d/a, respectively, with a rather decreasing trend.

Briefly, two relative high temperature indices including SU25 and TR20 had a definite increasing trend while two relative low temperature indices including ID0 and FD0 tended to decrease. Compared with both SU25 and ID0 based on daily maximum temperature, the trend of both TR20 and FD0 based on daily minimum temperature was more regular, indicating a definite increase and decrease, respectively (Fig 10c and 10d).

### 3.4 Temperature changes based on the disaster temperature index

#### 3.4.1 Temporal-spatial features.

The frequency of HTD40 was relatively high with the mean of 1.65d/a, and the regional differentiation was larger than that of LTD-10. The total frequency of seven stations during the studied period was 106d, 114d, 65d, 137d, 97, 127d, and 71d, respectively. The HTD40 of Qinyang, Boai, Xiuwu, and Mengzhou were higher—all exceeding 100d; those of the other three stations: Shanyang, Wuzhi, and Wenxian, were relatively lower. Furthermore, the average frequency of the former four stations was 55.79% higher than that of the latter three stations. More significantly, the frequency of Xiuwu (the highest) was 92.96% higher than that of Wenxian (the lowest).

Correspondingly, the frequency of LTD-10 was relatively low with the mean of 0.70d/a, and the regional differentiation was smaller than that of HTD40. The total frequency of seven stations during the studied period was 43d, 36d, 53d, 36d, 39d, 55d, and 51d, respectively. The LTD-10 of Mengzhou, Shanyang, and Wenxian were higher—all exceeding 50d; that of Boai, Xiuwu, Qinyang, and Wuzhi were relatively lower. The average frequency of the former three stations was 37.66% higher than that of the latter four stations. Rather strikingly, the frequency of Mengzhou (the highest) was 52.78% higher than that of Xiuwu (the lowest).

#### 3.4.2 Changing or variation conditions.

**Fig 11:**
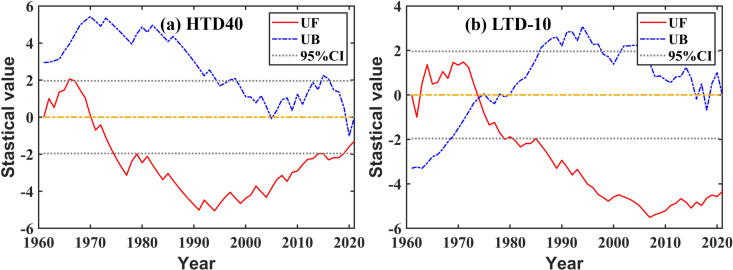
Mann-Kendall test for the disaster temperature index.

**Fig 12:**
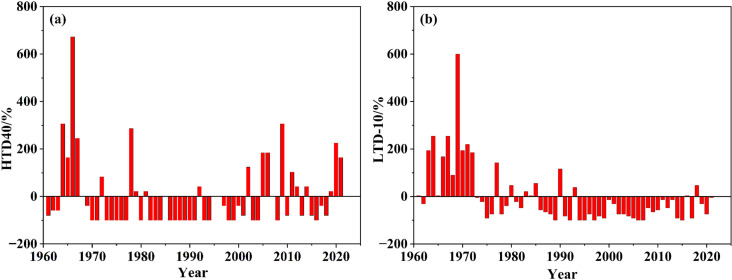
Processes and fluctuating ranges of the disaster temperature index.

Combined with Fig 11, there were two analysis-worthy points regarding the changing process of two disaster temperature indices in this article. At first, HTD40 fluctuated significantly while no obvious mutation occurred. There are two high values in the 1960s and the 2000s (Fig 12a). During the studied period for HTD40 > 10d, there were 11 years in the whole research region, and eight years during the 1960s and the 2000s—from 1964 to 1967, 2002, 2005, 2006, and 2009. From the 1960s to the 2010s for HTD40, the annual mean was 1.44d/a, 0.49d/a, 0.16d/a, 0.33d/a, 0.94d/a, and 0.74d/a, respectively. Generally speaking, the 1980s being the boundary, there was a decrease first, and then an increase.

Furthermore, there was a high-value period of the 1960s regarding LTD-10 and a low-value period of the 1990s with characteristics in sharp contrast to the fluctuating state regarding HTD40 (Fig 12b). Among them, the frequency of LTD-10 during 1969, 1964, and 1967 ranked the top three for the studied period, which were 81d, and double 41d, respectively. There were eight years during which the frequency of LTD-10 was 0, and four of them were during the 1990s: 1992, 1994, 1995, and 1997. From the 1960s to the 2010s, the annual mean of LTD-10 was 4.51d/a, 2.13d/a, 1.37d/a, 0.49d/a, 0.46d/a, and 0.97d/a, respectively, with an overall decreasing trend.

All in all, the frequency of HTD40 was high and the regional differentiation was large, while that of LTD-10 was low and the regional differentiation was small. Both the 1960s and the 2000s were the higher-value periods of HTD40, while 1960s was the higher-value period for LTD-10 and 1990s was the lower-value period for LTD-10.

## 4 Conclusion and discussion

### 4.1 Conclusion

Based on the basic data of daily minimum, maximum, and mean temperatures for the period 1961–2021 in Jiaozuo City, Henan Province, China, both the temporal-spatial features and the changing or variation processes of 14 temperature indices in four categories, including the mean temperature index, the extreme temperature index, the relative temperature index, and the disaster temperature index, were expressed by means of linear tendency estimation and anomaly fluctuation. Thereafter, this article presents the corresponding adaptive strategies on climate resource utilization to the main cultivated crops, according to regional temperature change trend. There are four main conclusions about regional temperature change:

(1) In terms of the mean temperature index, TMm had increased by 0.259 °C/10a, showing an obvious trend. For all stations, the years of the highest and lowest values were 2021 and 1984, respectively. Regional differences were large—Shanyang’s was the highest while Mengzhou’s was the lowest, and their difference exceeded 1 °C. Particularly, 2021 was not only a once-in-a-century flood year in the research region, but also the year with the highest annual temperature during the studied period.
(2) In terms of the extreme temperature index, TXm showed a fluctuation condition of first decreasing and then increasing, but TNm displayed an increasing trend that remained basically unchanged; the linear tendency rates were 0.200 °C/10a and 0.395 °C/10a, respectively. The diurnal range of DTR had a fluctuating trend, characterized by decreasing first and then increasing, taking approximately 1972 as a turning point.
(3) In terms of the relative temperature index, both SU25 and TR20 had been increasing while both ID0 and FD0 had tended to decrease. The relative high temperature indices in plain urban areas were larger while the relative low temperature indices in mountainous area were smaller.
(4) Regarding the disaster temperature index, HTD40 had a fluctuating feature of decreasing first and then increasing, taking the 1980s as the boundary, while LTD-10 showed a decline trend. Both HTD40 and LTD-10 had a same intergeneration of high-value period during the 1960s, while HTD40 had another high-value period during the 2000s and LTD-10 had a low-value period during the 1990s.

### 4.2 Discussion

The observed temperature changes in Jiaozuo City—particularly the increase in annual mean temperature, higher frequency of extreme high-temperature events (HTD40), and shifting seasonal temperature patterns—have direct and profound implications for the growth, yield, and management of major crops, particularly winter wheat and summer maize, which are dominant in this dual-cropping region. These climatic shifts are likely to curtail the growing periods, intensify heat stress during critical phenological stages, increase evapotranspiration demands, and alter pest and disease dynamics, thereby posing challenges to traditional cultivation systems. To effectively respond to these impacts and enhance the resilience of local agriculture, several adaptive strategies are proposed:

(1) In the context of continuous increase of annual temperature and the trend of higher daily maximum temperature, it is suggested that heat-resistant varieties be cultivated to alter the growth period of crops, and to achieve early sowing or late harvesting. For winter wheat, earlier sowing can help avoid late-spring heatwaves during flowering and grain filling stages. For summer maize, slightly delaying sowing may reduce exposure to early summer heat stress. Such adjustments will help crops better align with favorable thermal periods and avoid critical heat-sensitive phases. It will improve the yield and quality of crops.
(2) To cope with intensified heat stress, cultivation of heat-tolerant, drought-resistant, and early maturing varieties is essential. For winter wheat, varieties with brief growth cycles can avoid late-season heat. For summer maize, selecting cultivars with strong root systems and heat-resilient phenotypes will reduce the impact of temperature-induced water stress and ensure stable yield.
(3) In the context of the relevant high temperature index increasing and the relevant low temperature index decreasing, adjusting the planting structure is advisable. Specifically, expanding the sown area of maize and reducing early season summer crops can better match the warming, drying climate trend. Shifting more production into the autumn cropping window improves water-use efficiency and reduces heat stress risk.
(4) As increase in temperature induces higher evapotranspiration, irrigation strategies must adapt. The adoption of water-saving irrigation technologies (e.g., drip irrigation, sprinkler systems) and optimization of irrigation timing based on weather forecasts can enhance water use efficiency. Additionally, Conservation farming techniques such as mulching, reduced tillage, and organic matter input improve soil water retention and reduce thermal stress on crops. Appropriate fertilization schedules and weed control are also necessary to maintain crop vigor under heat stress conditions.
(5) In view of the variation trend characterized by increasing high-temperature disaster index and decreasing low-temperature disaster index, it is advisable to establish a regional system of meteorological disaster prevention or reduction of high-temperature disasters, to promote the development of an agricultural disaster-risk mechanism, and to make a significant effort to realize the transfer of agricultural disaster risk.

Although this study provides a comprehensive analysis of temperature trends and related climatic indices in Jiaozuo City, several limitations should be acknowledged. First, the research is geographically limited to a single representative area, which may not fully reflect the broader characteristics of other winter wheat and summer maize dual-cropping regions in China. Second, this study mainly focuses on temperature-related indices and does not incorporate other important climatic factors such as precipitation, humidity, solar radiation, and wind speed, which also have significant effects on crop growth and climate-induced agricultural risks. Third, socio-economic variables, technological progress, and changes in agricultural management practices were not considered; these factors can play critical roles in the effectiveness of adaptive strategies.

Future research should aim to extend the spatial scale of analysis to include multiple dual-cropping regions with varying geographic and climatic conditions. Integrating temperature data with other climatic variables and using crop simulation or climate impact models would facilitate a more holistic understanding of agricultural vulnerability. Furthermore, interdisciplinary approaches that combine climate science with socio-economic assessments and stakeholder input would help formulate more targeted and feasible adaptation strategies. These efforts will be valuable for enhancing the resilience of agricultural systems to climate change in China and beyond.

## Supporting information

S1 Data The Date. (XLSX)

## References

[1] Gaur A, Schardong A, Simonovic S. Effects of Global Warming on Precipitation Extremes: Dependence on Storm Characteristics. Water Resour Manage. 2018;32(8):2639–48. doi: 10.1007/s11269-018-1949-x

[2] Zhou BT, Qian J. Changes of weather and climate extremes in the IPCC AR6 (in Chinese). Clim Chan Res. 2021;17(6):713–8. doi: 10.12006/j.issn.1673-1719.2021.167

[3] IPCC. Climate Change 2013: the Physical Science Basis. Contribution of Working Group Ⅰ to the Fifth Assessment Report of the Intergovernmental Panel on Climate Change. Cambridge, United Kingdom and New York, NY, USA: Cambridge University Press; 2013.

[4] Cai W, Ng B, Wang G, Santoso A, Wu L, Yang K. Increased ENSO sea surface temperature variability under four IPCC emission scenarios. Nat Clim Chang. 2022;12(3):228–31. doi: 10.1038/s41558-022-01282-z

[5] Zou CN, Ma F, Pan SQ, Lin MJ, Zhang GS, Xiong B, et al. Earth energy evolution, human development and carbon neutral strategy. Petr Expl Devel. 2022;49(2):468–88.

[6] IPCC. Managing the Risks of Extreme Events and Disasters to Advance Climate Change Adaptation. A Special Report of Working Groups Ⅰ and Ⅱ of the Intergovernmental Panel on Climate Chang. Cambridge, United Kingdom and New York, NY, USA: Cambridge University Press; 2012.

[7] IPCC. Meeting Report of the Intergovernmental Panel on Climate Change Expert Meeting on Climate Change, Food, and Agriculture. Geneva, Switzerland: World Meteorological Organization; 2015.

[8] Luo Y, Tian YL, Dai M, Chen XM. Extreme temperature and precipitation events change features over Yunnan in recent 50 years and their relation with regional climate change (in Chinese). J Yunnan Univ (Nat. Sci.). 2015; 37(6): 870–7. doi: 10.7540/j.ynu.20140395

[9] Li S, Wheeler T, Challinor A, Lin E, Ju H, Xu Y. The observed relationships between wheat and climate in China. Agric Forest Meteor. 2010;150(11):1412–9. doi: 10.1016/j.agrformet.2010.07.003

[10] XIAO D-P, TAO F-L. Impact of climate change in 1981—2009 on winter wheat phenology in the North China Plain (in Chines). J Chin Eco-Agric. 2013;20(11):1539–45. doi: 10.3724/sp.j.1011.2012.01539

[11] XU J, LU Y. Meta-Synthesis Pattern of Analysis and Assessment of Earthquake Disaster System (in Chinese). Acta Geogr Sin. 2009;29(11):1–18. doi: 10.1016/s1874-8651(10)60080-4

[12] Christidis N, Stott PA. Attribution analyses of temperature extremes using a set of 16 indices. Weather and Climate Extremes. 2016;14:24–35. doi: 10.1016/j.wace.2016.10.003 PMC5351813 28344929

[13] Globalization, Climate Change, and Human Health. N Engl J Med. 2013;369(1):94–6. doi: 10.1056/nejmc1305749 23822792

[14] IPCC. Climate change 2021: The physical science basis. Cambridge, United Kingdom and New York, NY, USA: Cambridge University Press; 2021.

[15] IPCC. Climate change 2022: Impacts, adaptation and vulnerability. Cambridge, United Kingdom and New York, NY, USA: Cambridge University Press. 2022.

[16] Wang F, Duan K, Zou L. Urbanization Effects on Human-Perceived Temperature Changes in the North China Plain. Sustainability. 2019;11(12):3413. doi: 10.3390/su11123413

[17] Dong W, Li C, Hu Q, Pan F, Bhandari J, Sun Z. Potential Evapotranspiration Reduction and Its Influence on Crop Yield in the North China Plain in 1961–2014. Advances in Meteorology. 2020;2020:1–10. doi: 10.1155/2020/3691421

[18] Li J, Lei H. Impacts of climate change on winter wheat and summer maize dual-cropping system in the North China Plain. Environ Res Commun. 2022;4(7):075014. doi: 10.1088/2515-7620/ac814c

[19] Wang N, Wu J, Gu Y, Jiang K, Ma X. Factors Influencing the Spatiotemporal Variability in the Irrigation Requirements of Winter Wheat in the North China Plain under Climate Change. Agronomy. 2022;12(9):1987. doi: 10.3390/agronomy12091987

[20] Yin YX, Wang XJ, Ye ZW, Jiao SX, Pan X. Trend and probability characteristics of extreme maximum and minimum temperature in the Jiangsu Province from 1951 to 2013 (in Chinese). Res Envir in Yangtze Bas. 2018;27(6):1351–60. doi: 10.11870/cjlyzyyhj201806019

[21] Hao LS, Min XZ, Zhang WZ, Li CQ, Wei RJ. Impact of climate warming on winter wheat yield in Hebei Province (in Chinese). J Chin Agrometeor. 2009;30(2):204–7. doi: 10.3969/j.issn.1000-6362.2009.02.016

[22] Yu FS, Lian LS, Chu CC. Temporal-spatial variation characteristics of extreme temperature events in Shandong Province (in Chinese). Meteor Sci and Tech. 2017;45(5):843–50. doi: 10.19517/j.1671-6345.20160508

[23] Liu YT, Xu GL, Yin ZX, Hu CQ, Wang Y, Liao FQ. Spatio-temporal changes of surface air temperature in Anhui Province in the context of global warming from 1960 to 2014 (in Chinese). J Nat Res. 2017;32(4):680–91. doi: 10.11849/zrzyxb.20160459

[24] Wang L. Temporal and spatial variation characteristics of extreme temperature and extreme temperature events in 1961-2011 in Henan Province (in Chinese). Meteor & Envir Sci. 2013;36(2):31–6. doi: 10.16765/j.cnki.1673-7148.2013.02.010

[25] Zhao GY, Han Y, Liu MH, Xu C, Xu F, Hou JL. Spatial- temporal variation of extreme temperature events in Henan Province from 1961 to 2016 (in Chinese). J Xinyang Nor Univ (Nat Sci). 2019;32(1):95–101. doi: 10.3969/j.issn.1003-0972.2019.01.016

[26] Yüksel I. Global warming and renewable energy sources for sustainable development in Turkey. Renewable Energy. 2008;33(4):802–12. doi: 10.1016/j.renene.2007.05.040

[27] Field CB, Barros V, Stocker TF. Managing the risks of extreme events and disasters to advances climate change adaptation: special report of the intergovernmental panel on climate change. Cambridge: Cambridge University Press; 2012.

[28] Kong F. Spatial-temporal differentiation-based evolution characteristics of different extreme air temperature indexes in China from 1961 to 2018 (in Chinese). Wat Res & Hydr Engin. 2020;51(4):67–80. doi: 10.13928/j.cnki.wrahe.2020.09.004

[29] Patra A, Min S-K, Kumar P, Wang XL. Changes in extreme ocean wave heights under 1.5 °C, 2 °C, and 3 °C global warming. Weather and Climate Extremes. 2021;33:100358. doi: 10.1016/j.wace.2021.100358

[30] You Q, Kang S, Aguilar E, Pepin N, Flügel W-A, Yan Y, et al. Changes in daily climate extremes in China and their connection to the large scale atmospheric circulation during 1961–2003. Clim Dyn. 2010;36(11–12):2399–417. doi: 10.1007/s00382-009-0735-0

[31] Guan Y, Zheng F, Zhang P, Qin C. Spatial and temporal changes of meteorological disasters in China during 1950–2013. Nat Hazards. 2014;75(3):2607–23. doi: 10.1007/s11069-014-1446-3

